# T-shaped toothbrush for plaque removal and gingival health in children: a randomized controlled trial

**DOI:** 10.1186/s12903-022-02137-x

**Published:** 2022-04-07

**Authors:** Noraida Mamat, Shani Ann Mani, Mahmoud Danaee

**Affiliations:** 1 Present Address: Unit of Paediatric Dentistry, School of Dental Sciences, Health Campus, Universiti Sains Malaysia, Kubang Kerian, 16150 Kota Bharu, Kelantan Malaysia; 2 Department of Paediatric Dentistry and Orthodontics, Faculty of Dentistry, Universiti Malaya, 50603 Kuala Lumpur, Malaysia; 3 Department of Social and Preventive Medicine, Faculty of Medicine, Universiti Malaya, 50603 Kuala Lumpur, Malaysia

**Keywords:** Toothbrush, Plaque removal, Gingival health, Oral hygiene, Children

## Abstract

**Background:**

To evaluate an experimental T-shaped toothbrush for plaque removal and gingival health when compared to a conventional toothbrush among children.

**Methods:**

This single blind parallel randomized controlled trial was conducted on 8–10-year-old healthy schoolchildren with no history of recent antibiotic intake, proximal caries or more than 3 missing teeth per quadrant. A computer-generated randomization list allocated child to the 2 groups. Each child received detailed instructions for tooth brushing. Gingival health and plaque scores were recorded in school at baseline, 2 weeks, 1 month and 3 months in a portable dental chair by an examiner who was blind to the allocated toothbrush. A general feedback on the use of the T-shaped toothbrush was obtained at 3 months. Data was analyzed using two-way repeated measure ANOVA, Generalized estimating equation and Bonferroni test.

**Results:**

A total of 195 eligible children were invited, 110 parents gave consent and 100 children completed the study; 50 in each group. There were statistically significant reductions in mean gingival and plaque scores at each visit when compared to baseline for both toothbrushes (p < 0.05). There were no statistically significant differences between scores for the two toothbrushes at each visit (p > 0.05). Majority of participants gave positive feedback regarding the T-shaped toothbrush.

**Conclusions:**

Both toothbrushes had similar efficacy in removing plaque and improving gingival health among children. The T-shaped toothbrush is an alternative to the conventional toothbrush for oral hygiene in children.

*Trial registration* Retrospectively registered at ClinicalTrials.gov Registry—NCT03989479 18/06/2019.

## Background

Control of the biofilm is the prevention of caries, the most important measure being to disturb the biofilm mechanically using a toothbrush, while altering the tooth chemically, using a fluoride-containing toothpaste [1]. Effective toothbrushing allows routine removal of dental biofilm, thereby preventing its evolution into more pathogenic forms, reducing the risk for dental caries and gingivitis [2, 3]. Prevalence of dental caries remains high in developing countries with poor social and economic development [4, 5]. In Malaysia, 71.3% and 33.3% of preschool and school children experienced dental caries [6, 7]. The highest need in 12-year-olds was for periodontal care, with almost all children needing oral hygiene instruction [7]. Undoubtedly, preventive advice and services are crucial for future oral health programmes in Malaysia.

Efficient toothbrushing and satisfactory plaque control in children is hard to achieve despite comprehensive preventive programmes being in place [8]. There is no evidence that supervised toothbrushing is effective in caries control [9]. Additional challenges include inadequate information offered to parents [10], insufficient preventive services provided by dentists regarding toothbrushing and oral care instructions [11] and varied parent attitudes and practices among diverse socioeconomic groups [12].

Effective toothbrushing is dependent on patient compliance and manual dexterity of the child [13, 14]. Most children show poor compliance towards brushing because they consider it a tedious and repetitive procedure [14]. While older children are more adept at toothbrushing, their toothbrushing is still unsatisfactory [14]. Added factors influencing the effectiveness of toothbrushing in children include correct toothbrushing methods [15–17], frequency [18], duration [14] and toothbrush design [19, 20]. Toothbrushes that are more convenient to use, such as powered toothbrushes are more effective when compared to manual toothbrushes [21, 22]. However, powered toothbrushes may not be a feasible alternative for lower socio-economic groups [23].

Various modifications of manual toothbrushes were developed to enhance the mechanical removal of dental biofilm in children [19, 20, 24, 25]. The basic shape of manual toothbrushes has remained the same since the advent of modern toothbrushes. While minor changes in handle design, bristle material and arrangement within the same original shape have been studied extensively, the original toothbrush design has not changed significantly. Recently, a new toothbrush with an innovative T-shaped brush head (T-Toothbrush Denson™, Malaysia) was introduced, claiming it is designed to efficiently clean and reduce gingival inflammation [26, 27]. Unlike most conventional manual toothbrushes, this novel toothbrush was designed to predominantly use a vertical hand motion on tooth surfaces, suggesting that this allows the brushing process to be more controlled and stable [26]. A pilot study demonstrated that T-shaped toothbrush was accepted by 8–10-year-old children and significantly decreased plaque accumulation and improved gingival health in nineteen schoolchildren [28].

To date, no studies have evaluated the effectiveness of the T-shaped toothbrush in children in comparison to the conventional manual toothbrush. For this study we hypothesized that the T-shaped toothbrush is significantly better at removing plaque and maintaining gingival health in 8–10-year-old children when compared to the conventional manual toothbrush. The aim of this study was to evaluate the effectiveness of a T-shaped manual toothbrush in removing plaque (primary outcome) and maintaining gingival health (secondary outcome) when compared to a conventional manual toothbrush among school children aged 8–10 years.

## Materials and methods

### Study population and methodology

#### Study design

**Fig. 1:**
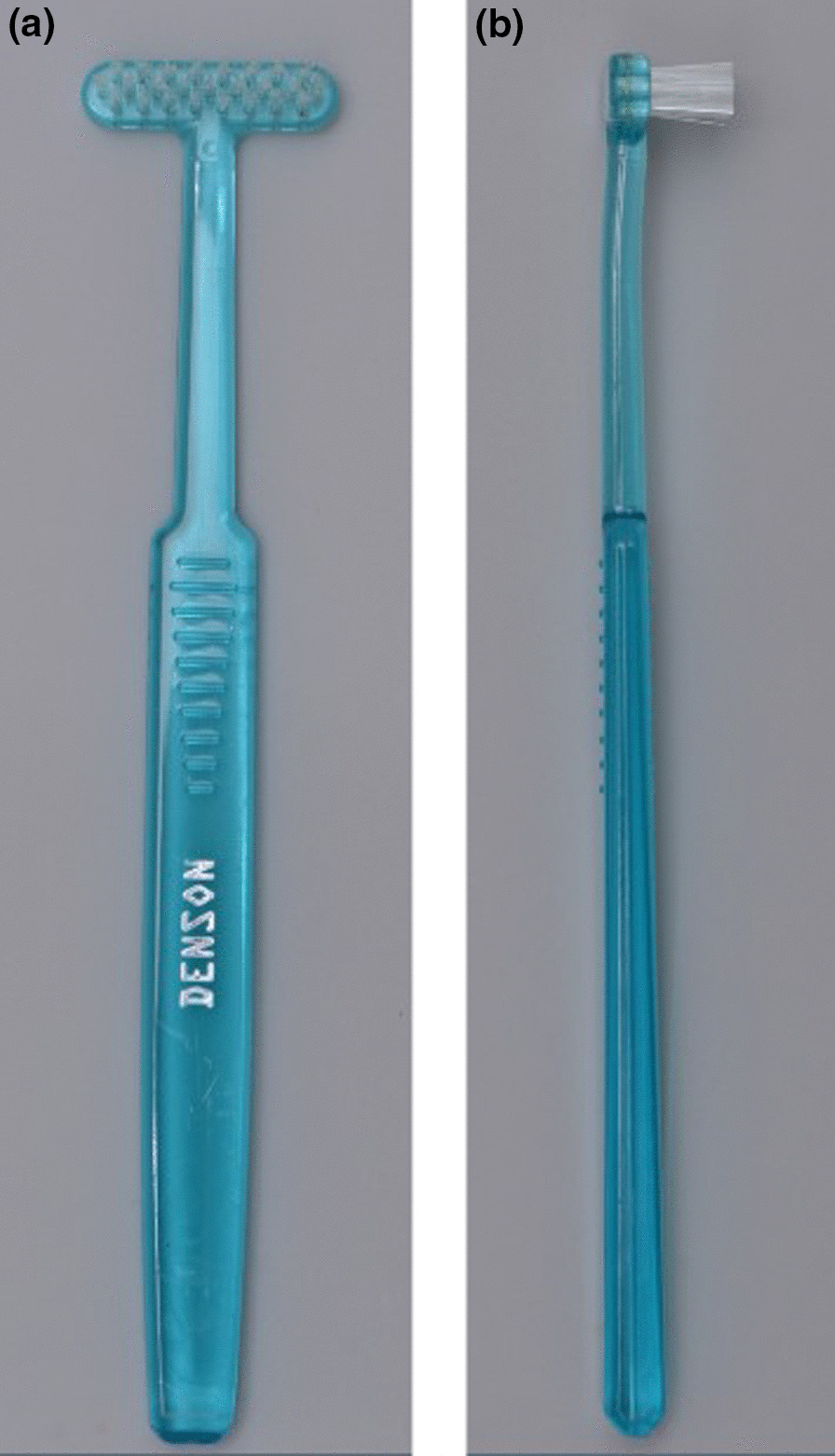
**a** and **b** Anterior and lateral view of brush head of T-shaped toothbrush (Denson™, Malaysia)

**Fig. 2:**
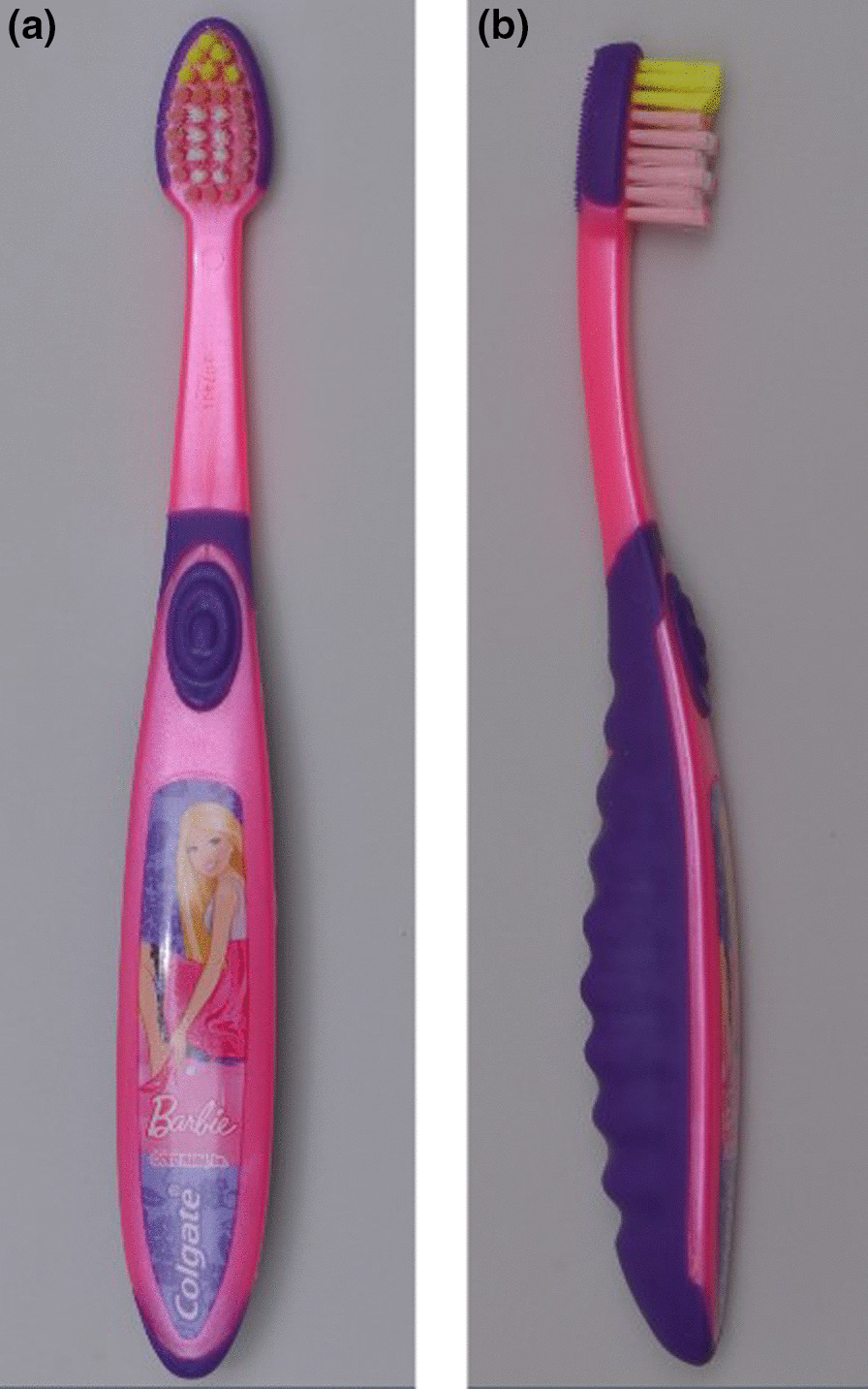
**a** and **b** Anterior and lateral view of brush head of conventional toothbrush (Kid’s Soft Toothbrush, Colgate)

This was a single blind, parallel randomized controlled trial (1:1 ratio) to compare the effectiveness of two manual toothbrushes; the T-shaped toothbrush (T-Toothbrush Denson™, Malaysia) (Fig. 1a, b) and a conventional toothbrush (Colgate® Kids Soft Toothbrush-Age 5–9 years) (Fig. 2a, b) among 8–10-year-old children.

#### Ethical approval and sample size calculation

Ethical approval was obtained from the Medical Ethics Committee, Faculty of Dentistry, University of Malaya, Kuala Lumpur [(Reference Number: DF CD1416/0093(P)]. All procedures performed were in accordance with the ethical standards of the institutional committee and with the 1964 Helsinki declaration and its later amendments or comparable ethical standards. In addition, necessary permission was obtained from authorities at Ministry of Education to conduct the study among children attending three public schools. The trial was retrospectively registered at ClinicalTrials.gov no. NCT03989479 on 18/06/2019.

The sample size was calculated based on the study by Rosema et al. [29]. We set an alpha error at 0.05 and power at 80%, where the clinically relevant difference of plaque index between the two groups was at 0.1 with standard deviation of 0.2. A sample size of 41 subjects was needed for the study. Considering an average 20% drop-out rate, the target sample size was 50 children in each group, to make a total of 100 subjects.

#### Subjects

Hundred ninety-five participants aged 8–10 years from three public primary schools who fulfilled the inclusion criteria were invited to participate in this study. Children were included if they had good systemic health, normal motor and cognitive development. Those who had acute intraoral lesion, history of antibiotic and/ or antiseptic therapy in the past one-month, history of recent visit to dentist or prophylaxis, interproximal caries or restorations and 3 or more missing teeth in one quadrant were excluded. Informed consent was obtained from the parents or legal guardians of all children included in the study.

### Data collection procedures

#### Conduct of the study

Children whose parents consented were allocated to the control (Colgate® Kids Soft Toothbrush-Age 5–9 years) and experimental group (T-Toothbrush Denson™, Malaysia) using a computer-generated randomization list based on the inclusion and exclusion criteria listed above. The allocation of groups was carried out by the study coordinator who ensured allocation concealment with participant information in numbered sealed envelopes. The children were clinically examined at baseline (Baseline) for plaque scores and gingival scores by one examiner (NM) on the school premises in a mobile dental chair with a portable spotlight. The gingival status was assessed first by probing gently along the wall of soft tissue of the gingival sulcus to determine the gingival score. Then, a plaque disclosing solution (Mira-2-ton, Hager Werken, Germany) was applied with cotton pellet to the teeth for plaque score measurement. After completing the clinical examination, each participant received instructions regarding the tooth brushing technique, handling and manipulation of the tooth brush individually by trained assistant (SL). A video depicting the brushing technique for each toothbrush was screened to the children followed by a hands-on demonstration on an enlarged teaching model of the mouth. The participants were then asked to brush immediately after the instructions were given. All participants were provided with a toothbrush and a tube of 1450 ppm fluoridated toothpaste (Fresh & White Refreshing Mint with Xylitol Toothpaste, Southern Lion) to be used twice daily for 2 min using the prescribed toothbrush and toothpaste (pea-sized amount) for a three-month period.

**Fig. 3:**
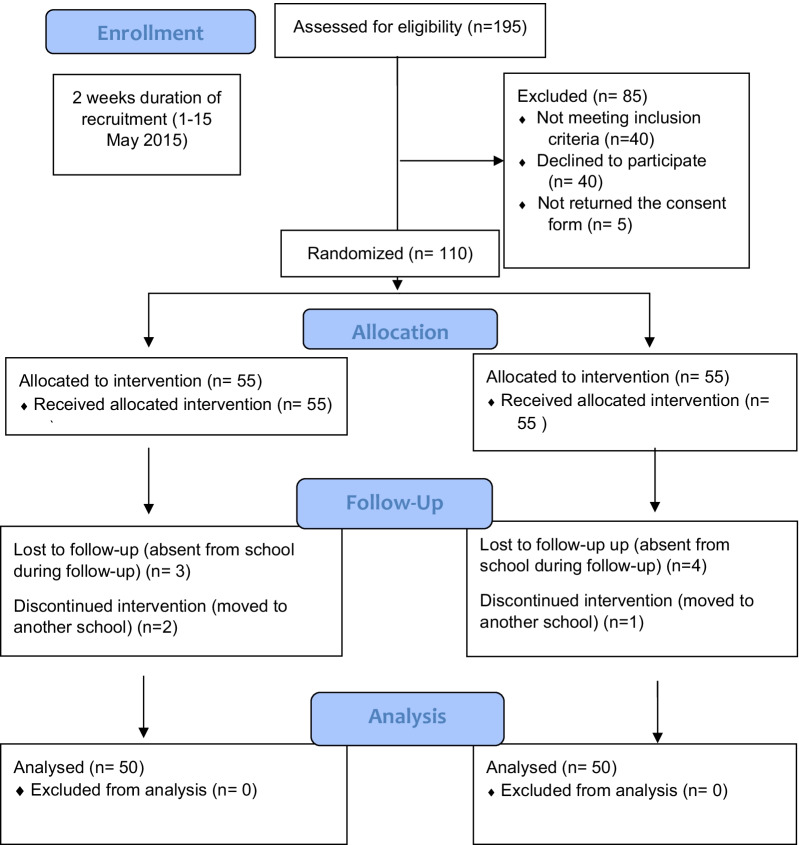
Flow of the participants during the study

Follow-up visits were done at 2 weeks (Visit 1), 1 month (Visit 2) and at the end of 3 months (Visit 3) for plaque scores and gingival scores by the same examiner (NM). Toothbrushing techniques were reinforced at each visit by the same trained assistant (SL) after clinical assessments were done. The participants were also instructed to refrain from using any other oral hygiene products or medication during the study period. No professional cleaning was performed prior to the start of the study. On the last visit (Visit 3), the children from the experimental group were also asked to answer a feedback questionnaire to assess the satisfaction after using T-shaped toothbrush [28]. The study was conducted between May 2015 and September 2015. The flow of the study is shown in Fig. 3.

### Toothbrush and technique of toothbrushing

#### T-shaped toothbrush (T-Toothbrush Denson™, Malaysia) (Fig. 1a, b)

The long axis of the head of the toothbrush was perpendicular to the handle. The head measured 31 mm x 8 mm. The bristles were flat trimmed, set in 3 rows, each row having 8–9 tufts and made from Dupont nylon 612. It had soft, round ending filaments of 1 cm length. The handle was straight, flat, rectangular shaped and made of hard plastic. The size of the handle was 111 mm x 13 mm. The scanning electron microscopy (SEM) image showed unevenly finished filaments with flat ends. The tufts were not well-defined and filaments in a tuft were splayed.

The vertical scrub technique was prescribed for all participants assigned with T-shaped toothbrush (T-Toothbrush Denson™, Malaysia). For all buccal surfaces, the toothbrush was held vertically with filaments placed perpendicular to the surfaces and the brush head was moved in an up and down motion (vertical direction). For the lingual and palatal surfaces, the toothbrush head was placed near the gingiva and moved vertically towards an occlusal direction. For the occlusal surfaces, the toothbrush was held horizontally and moved repeatedly in a back-and-forth movement (anterior posterior direction).

#### Conventional toothbrush (Colgate® Kids Soft Toothbrush-Age 5–9 years) (Fig. 2a, b)

The Kid’s Soft Toothbrush, Colgate (5–9-year-old) is a conventional toothbrush with brush head parallel and in line with the handle of the toothbrush. It had oval shaped head with size of 24 mm x 7 mm. The bristles were made from nylon, set in 4 rows with multi-levelled design. The toothbrush had extra soft and round ending filaments. The handle was curved and ergonomically designed comprising both hard and soft plastic components. The size of handle was 100 mm x 18 mm. The scanning electron microscopy (SEM) image revealed uniform rounded ended filaments arranged in well-defined tufts.

A modified Fones technique was prescribed for all participants assigned with the conventional toothbrush (Colgate® Kids Soft Toothbrush-Age 5–9 years). For all buccal and occlusal surfaces, the toothbrush was held horizontally with filaments placed perpendicular to the tooth surface, with part of the bristles on the gingiva and part on the tooth and activated in a circular motion. For the lingual surfaces, the toothbrush head was placed near the gingiva and moved in an occlusal direction.

### Indices

Gingival status was scored using the Löe and Silness modified by Löe, Gingival Index [30]. The amount of plaque was scored using the modified Quigley and Hein Plaque Index (TQHI) [31] Both indices were recorded at 6 sites around all the teeth (mesiobuccal, midbuccal, distobuccal, mesiopalatal/lingual, midpalatal/lingual, distopalatal/lingual). All teeth were included except teeth with crowns or cervical restorations.

### Inter and Intra-examiner reliability

The main examiner (NM) was blinded to the assigned groups. Calibration of the main examiner was done with one senior clinician (SAM) considered as gold standard, for both Löe and Silness modified by Löe, Gingival Index and modified Quigley and Hein Plaque Index (TQHI). The inter-examiner calibration was done on three children aged 8-to-10-years who attended the Paediatric Dentistry clinic, Faculty of Dentistry, University of Malaya. Full mouth scoring for each child was done by the paediatric dentist and the main examiner on one visit for the gingival score and plaque score. For intra-examiner calibration, the main examiner scored the gingival and plaque score on the same patient twice on the same day after a period of half an hour. Inter-examiner variability tested using intra-class correlation coefficient (ICC) was 0.96 and 0.88 for gingival and plaque score respectively. Intra-examiner variability for main examiner was 0.96 for gingival score and 0.89 for plaque score.

### Statistical analysis

The data was entered and analyzed using software package SPSS 25.0 [32]. Descriptive statistics such as frequencies and percentages (for categorical variables), mean, median standard deviation (for continuous variables) were calculated. The differences of total mean for gingival score and plaque score between visits within each group was assessed using two way repeated measure ANOVA for normally distributed interval dependent variable and GEE (generalized estimating equation) test for non-normal interval dependent variable. Mean comparison between and within groups was performed using Bonferooni test. Feedback regarding T-shaped toothbrush was analysed using descriptive statistics. In all analysis the level of statistical significance set at 5% (p < 0.05) with 95% confidence interval.

## Results

### Profile of study participants

**Fig. 4:**
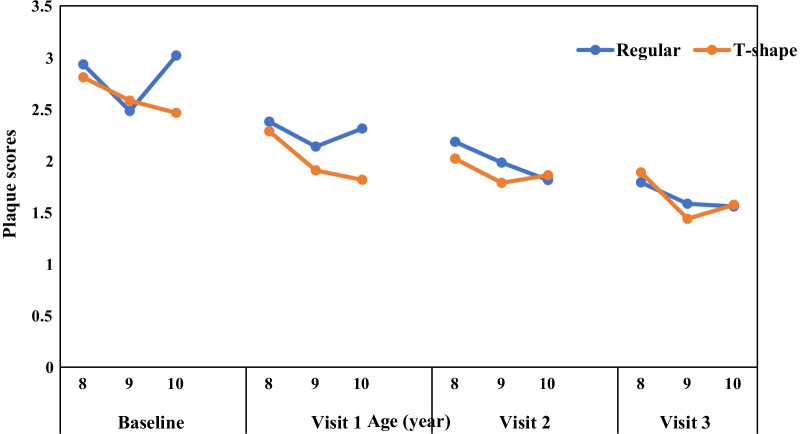
Differences in plaque score between age groups for both toothbrushes at each visit

A total of 110 children who fulfilled the inclusion and exclusion criteria were enrolled in the trial, however only 100 children successfully completed all three follow-up examinations (Fig. 3). The demographic characteristics of the participants are given in Table 1. Prior to data analysis the homogeneity between groups for sociodemographic and baseline plaque and gingival scores were examined and results showed both groups were not statistically different except for age. Since the age of two groups was significantly different, the relationship between age and both dependent variables were evaluated. There were differences in plaque scores between age groups for both tooth brushes at each visit (Fig. 4). Due to the small sample size for each age group, these differences were not statistically significant. There was no significant relationship between age and gingival score as well, therefore it was not considered as potential covariate.

**Table 1:** Profile of study participants

Variables	Control group (n = 50)	Experimental group (n = 50)	p value
	n (%)	n (%)	
Gender			0.316
Boy	24 (48%)	29 (58%)	
Girl	26 (52%)	21 (42%)	
Ethnicity			0.207
Malay	46 (92%)	41 (82%)	
Chinese	0 (0%)	1 (2%)	
Indian	3 (6%)	8 (16%)	
Other	1 (2%)	0 (0%)	
Age (in years)			0.019
8	13 (26%)	20 (40%)	
9	15 (30%)	21 (42%)	
10	22 (44%)	9 (18%)	
Plaque score at baseline	2.83(0.70)	2.65(0.95)	0.279
Gingival score at baseline	0.23(0.17)	0.20(0.15)	0.387

### Comparison of clinical parameters

**Fig. 5:**
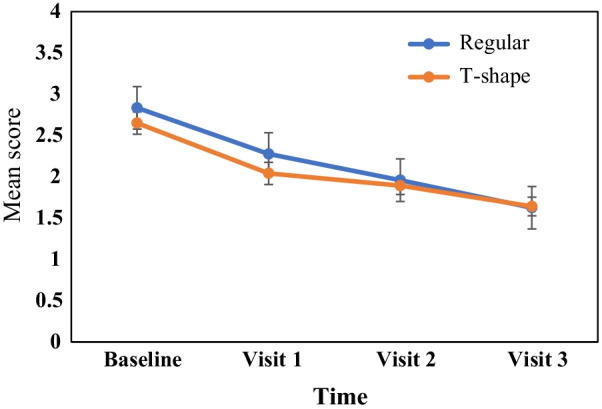
Mean plaque score for both regular and T shaped toothbrushes across time

**Fig. 6:**
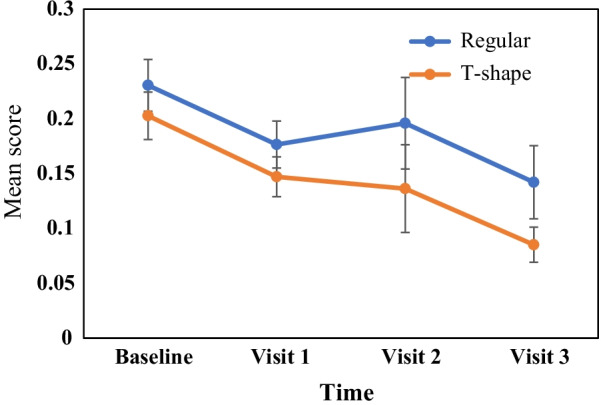
Mean gingival score for both regular and T shaped toothbrushes across time

There was a decrease in mean plaque and gingival scores from Baseline to Visit 3. Figures 5 and 6 show the mean plaque and gingival scores over time from Baseline to Visit 3 respectively.

Prior to data analysis plaque score were subjected to normality test and results showed this variable was normally distributed, therefore a two-way repeated measure ANOVA was employed to compare the plaque score between and within groups. The findings for within subjects’ effect (visit) of repeated measures ANOVA was significant (F _(2.5, 245.7)_ = 76.543. p < 0.001, η^2^ = 0.439) and revealed almost a large effect of time on plaque score in both groups. The results indicate that the interaction between group and time was not statistically significant (F _(2.5, 245.7)_ = 1.118. p = 0.342, η^2^ = 0.011) indicating that the changes of plaque score between both groups were not significantly different across the times (visits). The main effect of group (type of toothbrush) was not statistically significant (F _(1, 98)_ = 0.817, p = 0.368, η^2^ = 0.008).

Since the gingival scores were not normally distributed in both groups, GEE analysis was applied to assess whether there was significant difference for gingival scores between groups and within times. Regarding the results for the gingival scores, it was found that there was no significant difference between the groups (χ^2^ = 2.178, p = 0.140). In addition, there was a significant effect over time on gingival score (χ^2^ = 39.566, p < 0.001). The interaction between time and group was not significant (χ^2^ = 0.677, p = 0.879). To test the related hypothesis, post hoc test (Bonferroni) was applied to compare the mean scores within and between group changes (Tables 2 and 3). Results for between groups comparison (Table 2) indicated that there is no difference between two types of toothbrushes. Results for within groups comparison across visits (Table 3) indicated that there is significant reduction on plaque score and gingival score in both types of toothbrushes.

**Table 2:** Pairwise comparison of study groups across four visits for plaque and gingival scores

Variable	Time	(I) Type of toothbrush	(J) Type of toothbrush	Mean difference (I–J)	SE	p value	95% CI for difference	
							LB	UB
Plaque score	Baseline	Regular	T-shape	0.185	0.167	0.271	− 0.146	0.516
	Visit 1	Regular	T-shape	0.235	0.171	0.172	− 0.104	0.574
	Visit 2	Regular	T-shape	0.067	0.153	0.664	− 0.237	0.371
	Visit 3	Regular	T-shape	− 0.016	0.148	0.913	− 0.310	0.278
Gingival score	Baseline	Regular	T-shape	0.028	0.032	1	− 0.047	0.102
	Visit 1	Regular	T-shape	0.029	0.028	1	− 0.038	0.097
	Visit 2	Regular	T-shape	0.060	0.057	1	− 0.080	0.199
	Visit 3	Regular	T-shape	0.057	0.037	1	− 0.044	0.158

*I* regular toothbrush, *J* T-shaped toothbrush, *LB* lower bound, *UB* upper bound

**Table 3:** Pairwise comparison among visits for each group for plaque and gingival scores

Variable	Type of toothbrush	(I) time	(J) time	Mean difference (I–J)	SE	p value	95% CI for difference	
							LB	UB
Plaque score	Regular	Baseline	Visit 1	.559*	0.107	< 0.001	0.271	0.846
		Baseline	Visit 2	.876*	0.117	< 0.001	0.561	1.191
		Baseline	Visit 3	1.210*	0.126	< 0.001	0.872	1.548
		Visit 1	Visit 2	.317*	0.109	0.027	0.023	0.611
		Visit 1	Visit 3	.651*	0.103	< 0.001	0.375	0.928
		Visit 2	Visit 3	.334*	0.079	< 0.001	0.123	0.545
	T-shape	Baseline	Visit 1	.609*	0.107	< 0.001	0.322	0.897
		Baseline	Visit 2	.758*	0.117	< 0.001	0.444	1.073
		Baseline	Visit 3	1.009*	0.126	< 0.001	0.671	1.348
		Visit 1	Visit 2	0.149	0.109	1	− 0.145	0.443
		Visit 1	Visit 3	.400*	0.103	0.001	0.124	0.676
		Visit 2	Visit 3	.251*	0.079	0.011	0.04	0.462
Gingival score	Regular	Baseline	Visit 1	.0540*	0.014	0.003	0.010	0.098
		Baseline	Visit 2	0.035	0.044	1.000	− 0.066	0.135
		Baseline	Visit 3	0.088	0.036	0.299	− 0.021	0.198
		Visit 1	Visit 2	− 0.019	0.039	1	− 0.103	0.064
		Visit 1	Visit 3	0.034	0.031	1	− 0.042	0.111
		Visit 2	Visit 3	0.054	0.033	1	− 0.038	0.146
	T-shape	Baseline	Visit 1	.0556*	0.015	0.003	0.011	0.101
		Baseline	Visit 2	0.066	0.040	1	− 0.052	0.185
		Baseline	Visit 3	.1176*	0.018	< 0.001	0.061	0.174
		Visit 1	Visit 2	0.011	0.039	1	− 0.069	0.091
		Visit 1	Visit 3	.0620*	0.014	< 0.001	0.018	0.106
		Visit 2	Visit 3	0.051	0.031	1	− 0.035	0.138

*I and J* Time of study in pairwise comparison; *LB* lower bound, *UB* upper bound; * Significant difference at 0.05 level

### Feedback of the children regarding the use of T-shaped toothbrush

Overall, majority of participants (50–96%) gave a positive feedback regarding the T-shaped toothbrush in all aspects. Most of the children (70–74%) were comfortable with the shape and size of the brush head and only 16% children claimed it was difficult to use the toothbrush. In general, about 2% to 16% of the participants rated the T-shaped toothbrush negatively (Table 4).

**Table 4:** Satisfaction level of children regarding the use of T-shaped toothbrush

Question	Rating scales n (%)				
	Negative	Ok	Positive		
	Poor	Average	Good	Very good	Excellent
1. Clean feeling with tongue	3 (6%)	6 (12%)	4 (8%)	22 (44%)	15 (30%)
2. Clean feeling between teeth	0 (0%)	2 (4%)	16 (32%)	17 (34%)	15 (30%)
3. Clean feeling on back teeth	4 (8%)	6 (12%)	16 (32%)	9 (18%)	15 (30%)
4. Comfort during brushing	1 (2%)	5 (10%)	8 (16%)	3 (6%)	33 (66%)
5. Comfort after brushing	1 (2%)	4 (8%)	5 (10%)	9 (18%)	31 (62%)
6. Easy to use	8 (16%)	10 (20%)	13 (26%)	7 (14%)	12 (24%)
7. Shape of brush head	1 (2%)	14 (28%)	15 (30%)	4 (8%)	16 (32%)
8. Size of brush head	1 (2%)	12 (24%)	21 (42%)	13 (26%)	3 (6%)
9. Brush head enables reaching all areas	3 (6%)	2 (4%)	3 (6%)	20 (40%)	22 (44%)

## Discussion

The conventional manual toothbrush design has stood the test of time with very minor changes. While it is effective in plaque removal when used correctly, poorer dexterity of toothbrushing in children demands newer toothbrush designs which may be potentially easier to use. The T-shaped toothbrush is simple and a possible alternative for children [28].

We restricted the study age group to 8–10 years in order to limit the variability in dexterity and tooth brushing patterns between children. In addition, older children are more likely to brush for longer durations and more effectively [14]. In the present study, there were significant reductions in plaque and gingival scores at each visit when compared to the baseline values for both groups. This could be attributed to reinforcement of oral hygiene messages given at each visit. Similar findings were noted among 3 to 5-year-old children where regardless of the type of toothbrush, reinforcing regular oral hygiene instructions and practices were more important for plaque removal and improvement of gingival health [19]. The effectiveness of adequate oral hygiene instructions on the reduction of the plaque score and gingival score of children was also demonstrated in other studies [33, 34]. However, the outcome of a 2-year school-based toothbrushing study did not demonstrate significantly lower gingivitis and plaque scores in 8–11-year-old children, probably due to minimal reinforcement of toothbrushing and longer duration of study [35]. Evidently, the subjects in our study were compliant with the instructions. On the contrary, this may be attributed to the ‘Hawthorne effect’ and/or ‘Novelty effect’ [36], where subjects may modify or improve their brushing behaviour while being studied or observed in an experiment. Besides, the innovative appearance of the T-shaped toothbrush may stimulate children to be over enthusiastic in brushing more fervently and regularly.

From this study, there was no statistically significant difference between the two manual toothbrushes for plaque and gingivitis scores over a period of 3 months, although the regular toothbrush demonstrated a greater decrease. There were certain inherent differences between the two brushes that may have contributed to the results. The only available T-shaped toothbrush for children at the time of the study had a simple toothbrush handle with flat trimmed arrangement of bristles, while the conventional toothbrush had an ergonomic handle design and multilevel arrangement of bristles. Further, SEM images of the T-shaped toothbrush showed roughly finished round ended bristles with splayed tufts as compared to the conventional toothbrush which had smoothly finished round ended bristles with uniform tufts. Therefore, the conventional toothbrush had a certain advantage over the T-shaped toothbrush since scientific evidence suggests that multilevel arrangement of bristles [37] and ergonomic handles [38] boosts plaque removal efficacy. Consequently, the T-shaped toothbrush was apparently efficient regardless of the basic design and the novelty for children, who acclimatized well. Future studies should ensure that the tested toothbrushes are similar in design in all aspects such as bristle arrangements and handle design. Furthermore, we did not assess the ISO compliance of the T-shaped toothbrush, which has a significant implication on future marketability. Another reason for the lack of differences in gingival index between the toothbrushes could be the low gingival index value at baseline. Differences may have been apparent had there been evidence of gingivitis at baseline.

In the present study, the plaque score and the gingival status were assessed with routinely used indices for evaluating toothbrush efficacy [39] which fulfilled the criteria for an ideal index in clinical studies [40]. The scores were recorded on all teeth rather than on index teeth to obtain a more accurate result [39]. However, the modified Quigley and Hein plaque index did not measure the plaque accumulation on occlusal surfaces which has a predilection for caries, especially in children. During examinations, gingival index was recorded first to ensure better visualization of the gingival tissue followed by plaque index.

Brushing procedures and instructions were given during the first visit of the study. In contrast to the conventional toothbrush, the T-shaped toothbrush had to be used in a vertical direction. Consequently, participants were asked to brush immediately after the instructions were given, to be certain the children comprehended the toothbrushing technique. They were taught to brush in a systematic manner to ensure that all parts of the mouth and teeth were cleaned. In this study, clear brushing instructions was crucial to avoid the possibility of tissue trauma during brushing, especially if the T-shaped toothbrush was held in a horizontal manner while brushing the posterior teeth. Intensive individual training was essential to avoid the possibility of trauma and achieve the desired plaque control in the T-shaped toothbrush group.

Results from the feedback questionnaire on the use of T-shaped toothbrush revealed the potential for the toothbrush to increase motivation and improve compliance of the children towards regular habit of toothbrushing. The present study demonstrated acceptability of the T-shaped toothbrush. It was rated positively by majority of participants in all aspects related to cleaning, comfort and size or shape of the brush head.

This study is not without limitations. Firstly, the control toothbrush used in this study was more ergonomic and had different bristle arrangement when compared to the experimental toothbrush which had the basic design of a toothbrush. Ideally, both toothbrushes should have had similar parameters in terms of material, bristle arrangement pattern, cross-section of the bristle fields and design of the handle to be comparable, a recommendation for future studies. Thus, it is suggested that T-shaped toothbrush be made available with modifications such as rounded shape of the brush head, multilevel bristle arrangements and ergonomic handle that are comparable with other modern-day child toothbrushes. A study of a longer duration may also be able to differentiate the effect of the two brushes further and overcome the Hawthorne effect. Secondly, there was an advantage for the children in the control group since they did not have to adapt to a new toothbrush at the beginning of the study, as the toothbrush that was prescribed to them was most likely similar to their toothbrush at home. In contrast, the experimental group had a disadvantage wherein the children may have required some time to familiarize with the shape, vertical brushing technique and position of the brush handle of the T-shaped toothbrush. This drawback could have been overcome by a crossover design. Additionally, comparison of plaque removal between the buccal and lingual sides of the teeth, and between anterior and posterior regions would have further revealed the plaque removal efficacy of the new toothbrush. Children’s compliance of the modified fones technique for the control group is also questionable since they are more inclined to horizontal toothbrushing strokes rather than circular ones [8]. However, unlike Deinzer et al. [8], the children in this study were taught to do the lateral surfaces first using the circular motion and the occlusal surfaces last using the horizontal motion. Another limitation was that the children in this study were not monitored at home for their compliance of brushing with the prescribed toothbrushes. Parents were not informed of the details of the brushing technique and were not directly involved in the study. Consequently, there was a possibility that the children ceased using the T-shaped toothbrush at home, due to the novelty of shape and technique of brushing, which was entirely different from the conventional manual toothbrush. Parental motivation is important as children have a tendency to forget details and instructions [41]. Future studies should also encourage parental participation to ensure compliance of brushing using the prescribed toothbrush at home. Children’s compliance can be monitored using compliance calendar, diary sheets or recently introduced smart monitoring technologies where the participants or their parents record the toothbrushing events at home.

Further studies should explore the usefulness of T-shaped toothbrush on other age groups, especially in younger children where manual dexterity is limited. In addition, long term clinical studies for the efficacy of T-shaped toothbrush in controlling dental caries in children is needed.

## Conclusions

The results of this study indicate that there were no significant differences between the T-shaped toothbrush and the conventional toothbrush in terms of plaque removal and gingival health in 8–10-year-old children. The T-shaped toothbrush was able to remove plaque satisfactorily and maintain acceptable gingival health in children. This study provides the dental professional with scientific evidence on the effectiveness of the T-shaped toothbrush use in children, offering an effective option for oral hygiene in children.

## Data Availability

The datasets used and/or analysed during the current study are available from the corresponding author on reasonable request.
